# Determination of the Chemical, Physical and Mechanical Characteristics of Electric Arc Furnace Slags and Environmental Evaluation of the Process for Their Utilization as an Aggregate in Bituminous Mixtures

**DOI:** 10.3390/ma14040782

**Published:** 2021-02-07

**Authors:** Juan María Terrones-Saeta, Jorge Suárez-Macías, Evaristo Rafael Moreno-López, Francisco Antonio Corpas-Iglesias

**Affiliations:** Department of Chemical, Environmental and Materials Engineering, Higher Polytechnic School of Linares, Scientific and Technological Campus of Linares, University of Jaen, 23700 Linares, Jaen, Spain; jsuarez@ujaen.es (J.S.-M.); erml0001@red.ujaen.es (E.R.M.-L.); facorpas@ujaen.es (F.A.C.-I.)

**Keywords:** electric arc furnace slag, metallurgical industry, characterization, aggregate, bituminous mixture, mining waste, life cycle assessment, circular economy, sustainable construction

## Abstract

Road construction is an activity that demands a significant amount of aggregates for bituminous mixtures. In addition, these aggregates must be of a suitable quality for use, even more so on high traffic roads. In response to this problem, and in order to avoid the extraction of new raw materials, research is being carried out using industrial waste as a substitute for conventional aggregates. In this way, the extraction of raw materials is reduced and landfilling of waste is avoided. However, these wastes must have certain properties and environmental advantages over natural aggregates. Otherwise, the use of waste would not be environmentally beneficial but would be more damaging to the environment. For this reason, this research evaluates the viability of using electric arc furnace slag as aggregates for bituminous mixtures, the main objectives being the determination of the characteristics of the by-product, the particularities and the critical points to be taken into account for its subsequent use in mixtures. At the same time, the environmental advantages of treating this waste to obtain a usable aggregate are evaluated in comparison with the processing of a natural aggregate. The results showed that electric arc furnace slags have a suitable chemical composition and excellent physical and mechanical properties for use in bituminous mixtures, even on high traffic roads. At the same time, it was determined that their use produces a considerable reduction in environmental afflictions. Therefore, it could be affirmed that the use of electric arc furnace slags in bituminous mixtures is recommendable as a way to develop more sustainable materials for construction.

## 1. Introduction

The construction sector is one of the most demanding sectors for raw materials that exist today [1]. This sector produces a significant environmental impact associated with high greenhouse gas emissions [2,3]. These negative effects usually occur during the extraction phase of raw materials, as this is a sector in which a large quantity of these materials is used. The extraction of raw materials is generally a process that entails a significant environmental impact [4]. The extraction of aggregates seriously alters the landscape, also producing notable sound waves and seismic rays, as well as a series of by-products which are deposited in landfills [5]. In addition, these labors of extraction of raw materials have subsequent processes in which the material is adapted to obtain a shape and size of particle required by the regulations. Therefore, they induce treatments that consume significant amounts of energy to power machinery and, consequently, produce significant emissions of greenhouse gases and environmental effects [6]. Once these materials have been prepared for use in many finished products (concrete, fillers, bituminous mixtures, etc.) they must be transported to the place of use by various means of transport. These means of transport produce significant greenhouse gas emissions, as in most cases transport is carried out by road and with vehicles powered by fossil fuels.

Based on the above, it can be stated that the construction sector, which demands enormous quantities of raw materials, not only alters the final landscape through its exploitation, but also varies the image of the landscape from which the materials come. In addition, it emits significant greenhouse gas emissions in various locations and through different processes [7]. Therefore, and within a new circular economy [8] in which by-products are reused for new materials, it can be stated that the use of waste entails a reduction in environmental impact compared to traditional techniques [9]. However, it can be considered that waste has certain particularities (physical, mechanical or chemical) that must be studied in order to obtain final products of similar quality to conventional ones. In turn, the use of these industrial by-products does not avoid all the polluting phases of commercial products, but only some of them. Therefore, in order to quantify the effectiveness of the use of waste, its physical, chemical and mechanical properties must be analyzed, as well as the process and emissions that are produced by their use.

As mentioned, the use of waste is not only influenced by the processes that are eliminated and therefore do not produce environmental pollution, as can be the case with extraction of materials, but also by the quality of the by-product, since if the waste used does not provide the final material with the appropriate characteristics of durability, it will lead to a shorter working life of the product and, consequently, to the need for earlier renewal and greater pollution. Consequently, it is vital to study the physical, chemical and mechanical properties of the waste to confirm that it has similar properties to conventional materials [10] and, therefore, final materials with similar working lives will be obtained. If this were not the case, the reduction of environmental pollution, mainly caused by the activities of extraction of raw materials, would be limited by the continuous renovation of the material.

In short, the utilization of waste obviously produces environmental advantages [11,12]; however, its physical [13], chemical [14] and mechanical properties [15] must be evaluated to ensure that throughout the life cycle the reduction of greenhouse gas emissions and environmental impact is lower. Accordingly, in this research we evaluated the use of electric arc furnace slag as an aggregate for bituminous mixtures in roads [16]. This waste comes from the metallurgical industry of steel production. For its use, the physical, mechanical and chemical characteristics as well as the life cycle of the slag have been determined in comparison with conventional materials traditionally used with similar properties.

As mentioned above, electric arc furnace slag is derived from the process of producing steel from scrap [17]. This steel production process consists of two stages: a first stage called melting or primary metallurgy, in which the raw materials are melted; and a second stage called refining or secondary metallurgy, which is produced in the ladle furnace. The melting stage produces oxidation to remove the manganese and silicon in the raw material; this leads to defosphoration and the formation of electric arc furnace slag. In this slag all impurities are accumulated and it is the one used in this investigation.

Electric arc furnace slags have been used as building materials in different investigations, and even in real cases [18,19]. The electric arc furnace slags have been used as an aggregate for hot mix asphalt [20,21], warm mix asphalt [22] and even for mixes with bituminous emulsion [23]; this shows its excellent mechanical characteristics and its use in developing high quality bituminous mixtures [24]. However, in most cases the characteristics of this by-product have not been maximized and its use has been relegated to low-level materials [25].

On the basis of the above, in this investigation analysis electric arc furnace slag is proposed as materials for use in bituminous mixtures [26]. The choice of these slags for the proposed purpose is motivated by the importance of these aggregates in this material in a generalized way, by the relevance of this material in road infrastructures and by the very important demand for it in this type of infrastructure. For this purpose, and as a novelty with respect to other research relating to life cycle studies [27], the slag has been physically, chemically and mechanically characterized, thus determining its suitability, as well as the material it could replace within the bituminous mixtures. In this way, it has been possible to objectively evaluate whether its use entails the shortest working life of the final conformed product. At the same time, and with the aim to objectively evaluate the reduction of environmental impact [28] and greenhouse gas emissions from the use of waste, a life cycle analysis has been carried out comparing the results of the waste with those of commercial materials with similar characteristics. In other words, the electric arc furnace slag has been evaluated with respect to a similar quality aggregate used for coarse and fine aggregates in bituminous mixtures [29].

The life cycle analysis of the electric arc furnace slags was performed with the software SimaPro. Additionally, the physical, chemical [30] and mechanical tests established by the regulations for materials that conform the bituminous mixtures were carried out. The results reflected a clear reduction in the environmental impact by using this industrial by-product, as well as the presence of physical, chemical and mechanical properties suitable for using it in bituminous mixtures.

In short, this study not only quantifies the environmental advantages of using electric arc furnace slag as an aggregate for bituminous mixtures, but after the chemical, physical and mechanical characterization, the particularities of this material are obtained, as well as the critical points where special attention must be paid. Therefore, this research is crucial for all researchers who wish to develop various types of bituminous mixtures with electric arc furnace slag.

## 2. Materials and Methods

The purpose of this investigation is the assessment of the physical, chemical and mechanical properties of electric arc furnace slag as a coarse and fine aggregate for bituminous mixtures. In addition, and based on the properties obtained, the environmental pollution that the slag produces is compared with that produced by conventional and commercial materials of similar quality.

To do this, it is necessary to know the role that the waste plays within the bituminous mixture. Due to their characteristics, electric arc furnace slags are suitable as both coarse and fine aggregates.

Therefore, and with orders to follow an appropriate scientific methodology, firstly in the section on materials the origin of the by-product is defined, as well as its general characteristics and the treatment it received for its use. Subsequently, in the methodology section, the process to evaluate its physical, chemical and mechanical suitability for employment in bituminous mixtures is detailed, as well as the objective quantification of the environmental advantages of its use with respect to conventional or commercial aggregates of similar quality.

### 2.1. Materials

The material used in this investigation is a by-product of the metallurgical industry, electric arc furnace slag.

This material was taken unchanged from the producing company and then dried at a temperature of (105 ± 2) °C for 24 h. It must be noted that this process is carried out in order to eliminate humidity and therefore discard unnecessary variables that disturb the objective results of the methodology. Nevertheless, the existence of water in the material would not harm the industrial process, but it should be taken into account for the correct conformation of the final material.

In turn, it is necessary to specify that the physical, chemical and mechanical properties of the waste have been measured throughout time, that is, in different production batches. This fact is essential for the correct incorporation of a waste and the obtaining of a final material with acceptable properties, since, if on the one hand the properties of the waste were to vary throughout time (as happens, for example, with wastewater sewage sludge), its use would be unviable.

The subsequent sections describe the origin, production process and general characteristics of electric arc furnace slags.

#### Electric Arc Furnace Slags

The electric arc furnace slags, as detailed, come from the metallurgical industry of steel production, more specifically from the region of Andalucía, Spain.

These slags come from the first phase, called melting, of steel production from the use of scrap. In this phase, the steel is oxidized, dephosphorized and decarburized, forming a slag in which all the impurities are accumulated. It is called electric arc furnace slag. This slag is removed and cooled with water irrigation, so that in most cases it produces the hydration of chemical compounds that could produce problems of expansiveness.

The electric arc furnace slags, formed in the detailed process, possess some irregular shapes due to the aeration of the electric arc furnace from which it comes. In addition, electric arc furnace slags are produced in considerable quantities, with a production of approximately 150 kg per ton of final steel.

### 2.2. Methodology

Once the provenance of the electric arc furnace slags has been determined, we must proceed to define the methodology followed to evaluate the suitability of the by-product for use in bituminous mixtures, as well as to determine which conventional materials have similar properties.

It is therefore essential to quantify the physical, chemical and mechanical properties of the slags with different tests. Subsequently, and through the SimaPro software version 8.3.0.0 from PRé Consultants (Amersfoort, The Netherlands), the environmental advantage that the use of the above-mentioned by-product represents with respect to the commercial materials of similar quality is determined objectively.

Finally, and according to the results obtained in the previous sections, the use of electric arc furnace slag as materials for bituminous mixtures is objectively compared, elucidating the environmental advantages of such reuse.

To define in greater depth the methodology followed, and with the order to ensure the reproducibility of the results, the following sections describe the tests carried out on electric arc furnace slags, as well as the methodology of life cycle analysis.

#### 2.2.1. Characterization of the Electric Arc Furnace Slags (EAFS)

Waste characterization, as detailed above, is essential to evaluate the suitability of by-products. This characterization must be complete in determining the essential properties of the materials, as well as those critical points that, even when not harmful to the final material, must be taken into account in order to obtain an acceptable product.

To do this, we initially proceeded to the chemical characterization of the electric arc furnace slags. The chemical analysis consisted of different tests, the first of which was an elemental analysis. This test allowed us to quantify the percentage of hydrogen, nitrogen, carbon and sulfur in the sample by the combustion of the same. The test was performed with LECO’s TruSpec Micro commercial equipment (TruSpec Micro, LECO, St. Joseph, MI, USA). In addition, and given that the by-product analyzed is potentially inorganic, the X-ray fluorescence test was carried out to quantify the percentage of the various chemical elements with the highest atomic weight. The X-ray fluorescence test was performed with the commercial equipment ADVANT’XP+ (ADVANT’XP+, Thermo Fisher, Waltham, MA, USA). However, the properties of materials are not influenced by the presence of some chemical elements or others only, but mainly by the chemical compounds in which these elements are found combined. In the aim of determining the chemical compounds existing in the electric arc furnace slags, the X-ray diffraction test was carried out. This test was carried out with the equipment model X’Pert PRO of the commercial brand PANalytical (X’Pert PRO, PANalytical, Malvern, UK).

After determination of the chemical composition of the electric arc furnace slag, we proceeded to determine the physical properties. Firstly, in order to evaluate the surface of the particles, the scanning electron microscope test was carried out. This equipment, through high magnification, allows the microscopic image of the surface to be observed, thus obtaining essential qualitative information that will greatly condition the behavior of the slag. For this purpose, the dry slag, according to the procedure detailed above, was sieved through the 0.125 mm sieve to obtain the test sample. The scanning electron microscope used was a high resolution (FESEM), MERLIN (Carl Zeiss, Oberkochen, Germany) with EDX and WDX (Oxford Analytical, High Wycombe, United Kingdom) capabilities.

The electric arc furnace slags were tested by particle density according to the UNE-EN 1097-6 standard, to determine whether volumetric corrections are necessary for a density different from that of a conventional aggregate. The following tests were performed: the water absorption test according to UNE-EN 1097-6, to quantify the increased bitumen absorption in bituminous mixtures and the susceptibility to thermal fatigue; the sand equivalent test according to the UNE-EN 933-8 standard, to determine the presence of colloidal particles; the broken surface test according to the UNE-EN 933-5 standard and the flakiness index test according to the UNE-EN 933-3 standard, to determine the shape of the particles and their suitability for use in bituminous mixtures. For the purpose of determining the resistance of the electric arc furnace slags, since this by-product will be used as a coarse and fine aggregate in bituminous mixtures, the resistance to fragmentation tests were carried out in accordance with the UNE-EN 1097-2 standard, to qualify the hardness of the material; resistance to freezing and thawing cycles tests were performed (standard UNE-EN 1367-1), to evaluate the resistance to thermal fatigue of the aggregate; and determination of the polished stone value tests were performed (standard UNE-EN 1097-8), to quantify the resistance to continuous friction of the tire.

Finally, and given that electric arc furnace slags were produced in steel production, and thus come from processes where the existence of heavy metals are common as the leachates from these slags, we proceeded to the analysis of the same with the aim to determine that their use does not lead to significant environmental problems. To this end, a leaching test was carried out in accordance with the UNE-EN 12457-3 standard, detecting the presence of a high proportion of contaminating elements, comparing them with the limits set by Spanish regulations and limiting their use for the proposed purpose. For the analysis of the leachate, the commercial equipment Agilent 7900 (7900, Agilent, Santa Clara, CA, USA) was used.

#### 2.2.2. Life Cycle Assessment of the Electric Arc Furnace Slags in Comparison with Conventional Aggregates

The objective of this section is the evaluation of the environmental benefits produced by the use of electric arc furnace slags, as coarse and fine aggregate, for bituminous mixtures in comparison with conventional aggregates of similar quality.

To this end, once the physical, chemical and mechanical properties of the electric arc furnace slag were evaluated for its suitability for utilization, we proceeded to evaluate the environmental benefits which result from its use. For this purpose, the SimaPro software version 8.3.0.0 from PRé Consultants (Amersfoort, The Netherlands) was used.

An evaluation of the life cycle was carried out on two different materials, electric arc furnace slags and siliceous aggregate of similar quality. This methodology is regulated by the ISO 14040 and ISO 14044 standards. Therefore, it is necessary to study the different stages in both materials in order to obtain gravel and sand that can be used in bituminous mixtures. These stages are detailed below:

- Alteration of the landscape, geology and hydrogeology. The work of extracting materials is very polluting. In addition, the extraction directly from the physical environment produces a series of environmental impacts in the area of extraction which must be considered. Firstly, and as a previous task, any type of vegetation must be eliminated and a suitable terrain must be provided for the extraction of aggregates. This stage, therefore, has a significant influence on the vegetation and fauna, as well as on the various ground water and surface water flows.
- Raw material extraction. The various tasks involved in extracting the material produce a significant environmental impact on the physical environment in which they are carried out. Firstly, the continuous transport of machinery and the creation of roads for this equipment have an impact on greenhouse gas emissions and fauna. At the same time, it is usual to use explosives to obtain smaller fragments that can be treated for the manufacture of aggregates. These explosives produce a series of afflictions on the environment such as: seismic waves, air waves and dust. All these materials, after blasting, must be loaded by equipment that consumes fossil fuels in large trucks to be transported to aggregate processing plants, with the consequent emission of greenhouse gases.
- Freight transport. Vehicles with a high loading capacity and powered in most cases by fossil fuels transport the material loaded in the previous stages to the aggregate processing plant. This transport, through different routes made for the vehicles, produces a series of considerable greenhouse gas emissions. Therefore, for this study, a distance of 20 km has been taken as the distance between the extraction site and the aggregate processing plant. This distance is limiting in aggregate processing plant.
- Aggregate processing. Aggregates produced in quarries have very variable dimensions. There are large blocks of dimensions that are not acceptable for bituminous mixtures and even very small particles. Therefore, it is essential to process these materials in order to obtain a classification according to particle size. With the particle size classification, bituminous mixtures can be made by combining different stockpiles. As mentioned, the work of processing aggregates takes care of size reduction and classification according to particle size for their benefit in bituminous mixtures. This essential stage produces the final marketable material, and is therefore considered the last phase.

Once the phases into which the life cycle analysis is divided have been defined, the methodology followed is determined. It should be noted that the cradle-to-door life cycle analysis is to be executed until an aggregate is obtained that can be used for bituminous mixtures, whether from electric arc furnace slags or conventional aggregates. Subsequent phases of transport, manufacture of bituminous mixtures, spreading and compacting are not taken into account in this research for various reasons. On the one hand, there is a diversity of bituminous mixtures, so it would be unfeasible to analyze all of them. On the other hand, each bituminous mixture is manufactured with different binders and processes. Finally, the variations in the conformation of bituminous mixtures with electric arc furnace slags or conventional aggregates are minimal, since the greatest environmental difference is found in the stages defined above.

The methodology of calculation followed was CML 2000 in its version 2.05 (Centre for Environmental Studies, Leiden, the Netherlands). This methodology was followed for different reasons which are detailed below.

- On the one hand, there is a diversity of publications in various fields that have used this type of methodology and that have obtained adequate results.
- On the other hand, it possesses a high versatility, identifying different environmental factors that are affected by one process or another.
- Finally, the values obtained are based on statistics referenced at a European and even world level. Therefore, the extrapolation of the results is feasible in several countries.

Furthermore, this type of methodology analysis includes different factors such as: abiotic depletion, acidification, eutrophication, global warning potential, one-layer depletion, human toxicity, fresh water aquatic ecotoxicity, marine aquatic ecotoxicity, terrestrial ecotoxicity and photochemical oxidation.

At the same time, and in order to carry out this methodology, different reliable data have been used and corroborated by various researchers in the life cycle inventory. These data come from various sources:

- Empirical data measured directly from the industries producing road aggregates.
- Bibliographic data from various authors worldwide and in Europe.
- Different prestigious databases such as Ecoinvent v.3.2 (Ecoinvent, Zurich, Switzerland).

Once the software, the environmental inventory data and the methodology followed had been determined, the results of the environmental condition were obtained for the two scenarios evaluated. These scenarios were, as detailed above, on the one hand, the evaluation of the environmental cost of the production of usable aggregates in bituminous mixtures for roads with siliceous rocks. On the other hand, the use of electric arc furnace slags for aggregates in bituminous mixtures. Both scenarios were compared and partial conclusions were reached on the use of electric arc furnace slags. In this way, not only the physical, chemical and mechanical properties detailed above were objectively evaluated, but also the environmental benefits of using electric arc furnace slags as aggregates for bituminous mixtures.

## 3. Results

This section first describes the results of physical, chemical and mechanical tests to check the validity of electric arc furnace slags as coarse and fine aggregates for bituminous mixtures. At the same time, and in order to determine the environmental savings produced by their use, a life cycle analysis is carried out. This life cycle analysis compares the environmental effects of electric arc furnace slag with those of a siliceous aggregate of similar quality.

### 3.1. Characterization of the Electric Arc Furnace Slags (EAFS)

Firstly, with the order to evaluate the characteristics of the electric arc furnace slags, chemical tests were carried out. These chemical tests evaluated the composition of the slag and determined the existence of different elements or compounds that condition its use. The first of the tests carried out was that of elemental analysis. The results of this test are reflected in Table 1.

Elemental analysis of the slag has reflected the low proportion of carbon and hydrogen in the slag. This fact was to be expected, as slag is an inorganic material that is produced at high temperatures. At the same time, it should be noted that the percentages of sulfur and nitrogen are very low. Therefore, it can be stated that there will be no subsequent pollution problems due to the leaching of these elements in water.

Subsequently, and in order to characterize the elemental composition of the electric arc furnace slag, the X-ray fluorescence test was carried out. This test reflected the elemental composition detailed in Table 2.

The X-ray fluorescence test showed an elemental composition of the electric arc furnace slags that was to be expected, due to their production process. There is a high percentage of calcium, since it is the element added in the production industry for the production of the slag and the treatment of the material. The same applies to aluminum and silicon. At the same time, high percentages of iron are observed, since the slags proceed from the treatment of the steel and the iron is the main element of this material. Appreciable percentages of magnesium, manganese and chrome are present in electric arc furnace slags, being common elements in the scrap used in the metallurgical industry. The other elements are found in very low proportions, so their influence on the physical and chemical characteristics will be negligible. However, leachate of these heavy metals can be produced in excess of regulatory limits. To quantify this matter, a leaching test is subsequently carried out and it is confirmed that the concentrations of the polluting elements are lower than the limits set by European regulations.

**Figure 1:**
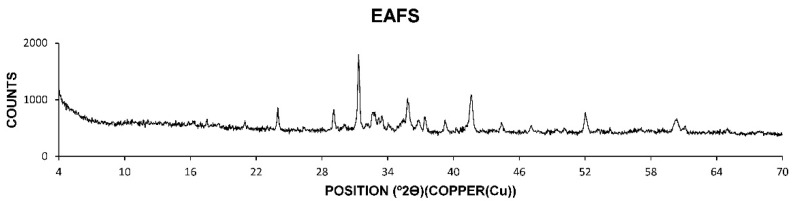
Results of X-ray diffraction of electric arc furnace slag.

It should be highlighted that the chemical elements have less or more activity depending on the compounds in which they are found combined. Therefore, the chemical composition is essential to calculate, as it will subsequently condition the physical and mechanical characteristics, as well as compatibility with other materials such as bitumen. The chemical composition of the slags was found through the X-ray diffraction test. The diffractogram obtained by the mentioned test, in a particular area where most of the compounds of this heterogeneous material are present, is shown in Figure 1.

**Figure 2:**
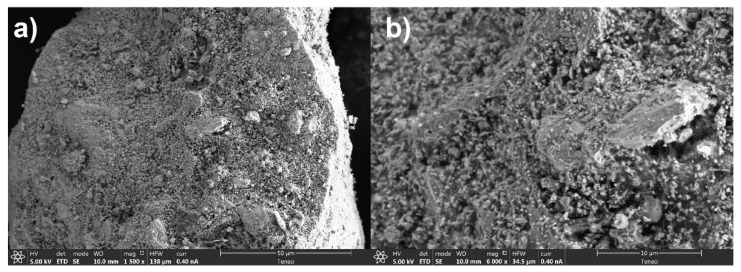
Images obtained of the electric arc furnace slags at different amplifications in the secondary option. (**a**) 1500×. (**b**) 6000×.

The diffractogram of the electric arc furnace slags shown in Figure 1 shows a high content of amorphous or non-diffracting material. Iron oxide and mixed iron and manganese oxides are identified as the main phases. Wollastonite is also identified in smaller proportion. This series of phases or chemical compounds is considerably stable, so that later problems of expansiveness, change of shape or composition due to hydration or carbonation of the chemical compounds are avoided. This fact ensures the stability of the slags and the correct chemical composition for their use as road aggregates. After analyzing the chemical composition of the slags, the physical properties were determined. The first of the tests carried out was the scanning electron microscope test. The images of the electric arc furnace slag obtained with the scanning electron microscope are shown in Figure 2.

Scanning electron microscope images at different amplifications mainly show the texture of a vitrified material. This fact corroborates the stability of the chemical compounds determined by the X-ray diffraction test. At the same time, an irregular surface can be seen that provides a micro-texture suitable for the grip of the tire on the pavement. Therefore, it ensures the slip resistance of the slag in bituminous mixtures. It is worth noting the existence of pores and micropores in the entire surface, so there is a greater specific surface and, consequently, a greater absorption of bitumen to be taken into account.

The following physical tests are the usual ones carried out for aggregates to be used in bituminous mixtures These tests are defined in Table 3.

**Figure 3:**
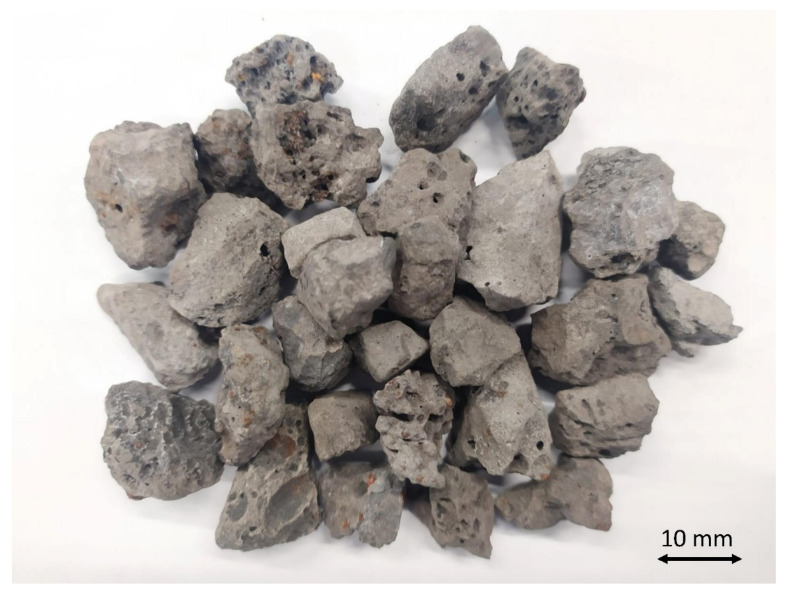
Image of electric arc furnace slags.

As can be seen in Table 3, EAFS have a higher particle density than a conventional aggregate (2.65 t/m^3^). This greater density is conditioned by the chemical composition of the slags, since there is a high percentage of iron and other heavy metals. This fact is not in itself a negative aspect, but it must be taken into account for the combination of the slag with other materials such as bitumen and to carry out an adequate proportioning. The water absorption test showed slightly higher values than for a conventional aggregate, mainly due to the slag formation process. This result most likely represents a higher bitumen absorption in the mix, which should not be negative, as well as a higher susceptibility to thermal fatigue. However, this latter characteristic was evaluated in the subsequent strength tests. The sand equivalent test shows a low percentage of colloidal particles, so it can be stated that there should be no subsequent problems of expansiveness or adhesion with the bitumen by this type of particle. The excellent shape of the electric arc furnace slag particles for use in bituminous mixtures dedicated to high traffic roads should be highlighted. This property is demonstrated through the broken surface and flakiness index tests. Both tests reflect a type of particle with similar dimensions in the three axes and with an infinite number of sharp edges; this characteristic can be seen in Figure 3. Therefore, the shape of the particles ensures a correct fit of the aggregates in the bituminous mixtures, as well as a high internal friction, allowing them to be used for mixtures with discontinuous grading and high traffic loads.

Finally, resistance tests will corroborate the mechanical properties of electric arc furnace slags for use as road aggregates. These tests are shown in Table 4.

The fragmentation resistance test of the electric arc furnace slags reflects the excellent mechanical properties of the slags. This result therefore ensures that there will be no subsequent breaking of the slags and consequently no compaction of the bituminous mix due to the constant passage of vehicles. On the other hand, and due to the fact that the image of the scanning electron microscope showed a surface of the slag with high percentages of pores, the test of resistance to the freezing and thawing cycles was carried out. This test showed an excellent resistance of the slag to thermal fatigue. Finally, it should be recalled that the slags had an adequate surface to ensure the friction of the tire with the pavement. This surface must be maintained over time, this factor being evaluated through the test of polished stone value. This test has shown that the slag from the electric arc furnace has a high resistance to the continuous passage of vehicles and that its micro-texture characteristics are preserved over time.

Finally, and with the order to determine that the electric arc furnace slags do not have problems in the production of polluting leachate, the leaching test was carried out as shown in Table 5.

The chemical composition of the electric arc furnace slags reflected the existence of different heavy metals. Therefore, in order to avoid environmental pollution from the use of the slags in bituminous mixtures and their contact with water, the leaching test was carried out. The concentrations of the polluting elements shown in Table 5 are lower than the limits established by Spanish regulations. Consequently, it can be assured that no polluting leachate will be produced; even less so, when these slags are enveloped by the bitumen and make their leaching into the bituminous mix difficult.

In short, and according to chemical, physical and mechanical tests, it can be stated that the electric arc furnace slags assessed possess suitable properties for use in road bituminous mixtures. The mechanical and textural properties of the slag also confirm that they are suitable even for high traffic roads.

### 3.2. Life Cycle Assessment of the Electric Arc Furnace Slags in Comparison with Conventional Aggregates

Once the physical, chemical and mechanical suitability of electric arc furnace slags for use in bituminous mixtures for roads had been confirmed, the environmental benefit of their use was evaluated with respect to a siliceous aggregate of similar quality. It should be noted that the slag was compared with a siliceous aggregate because it is this type of rock which provides greater resistance and therefore has similar mechanical properties. In turn, it should be mentioned that the life cycle analysis is evaluated up to the production of different stockpiles of aggregates of different particle sizes, whether they are slags or conventional aggregates. The subsequent stages of conforming, transporting, spreading and compacting of bituminous mixtures do not differ much between the waste and the conventional material, so they have not been considered in this research.

**Figure 4:**
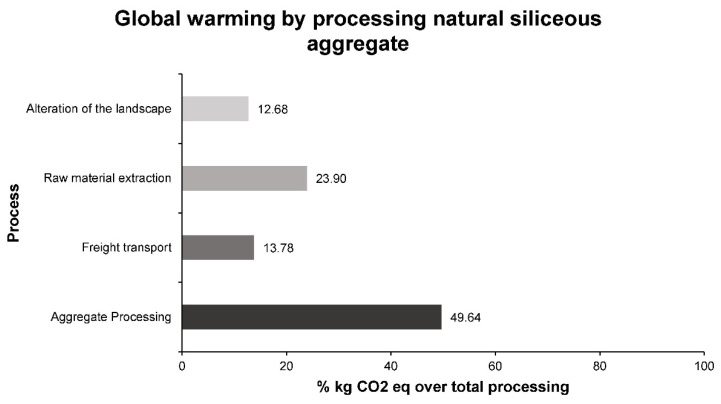
Percentages of kg of CO_2_ per stage in the process of obtaining natural silica aggregates for use in bituminous mixtures.

Firstly, the environmental impact of the production of 1 ton of natural siliceous aggregates with different particle sizes, coarse and fine aggregates was analyzed. Among the different impacts, the most significant is the one related to climate change, called global warming. This impact, measured in kilograms of CO_2_ equivalent, represents the emissions produced in the different stages of the process to obtain aggregates that can be used in bituminous mixtures. In order to evaluate which stage produces the greatest environmental impact within the detailed process, Figure 4 shows the percentages of impact of each stage on the total impact of the process. The kilograms of CO_2_ equivalent to the entire process reflect a value of 6.043.

As can be seen in Figure 4, the stage that produces the greatest impact, according to the global warming factor, is the processing of aggregates. This was to be expected, since in this phase large crushing equipment is used, fed by conveyor belts and occupying considerable space. This stage is common for electric arc furnace slags and natural siliceous aggregates, so the impact associated with it will remain even when using an industrial waste. On the other hand, the material extraction stage also has an important impact, as this stage involves the blasting of the rock and its fractionation into smaller blocks, and then, by means of equipment powered by fossil fuels, the loading of the vehicles that will transport them. The freight transport stage carried out in large vehicles has a similar environmental impact to the alteration of the landscape. Both factors must be taken into account in order to reduce the environmental impact caused by this process.

**Figure 5:**
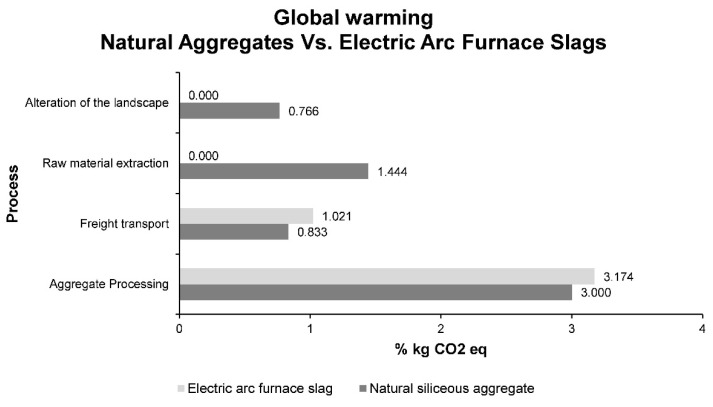
Global warming emissions in kilograms of CO_2_ equivalent for electric arc furnace slags and natural siliceous aggregates.

Subsequently, in order to compare the impact of global warming, in Figure 5, measurements of kilograms of CO_2_ equivalent are shown for each of the process phases and for the two materials: electric arc furnace slag and natural siliceous aggregates.

Figure 5 shows how the emissions due to the alteration of the landscape, geology and hydrogeology by the electric arc furnace slags are null. This is due to the fact that electric arc furnace slag is an industrial by-product and therefore is produced without altering the landscape. Similarly, emissions from the extraction of raw materials in electric arc furnace slag are null, as it is not necessary to extract it because it is produced as a by-product of the metallurgical industry. On the other hand, it can be seen that CO_2_ emissions are higher in the freight transport stage in electric arc furnace slag than in natural siliceous aggregates. This is due to the fact that the slag has a higher density, therefore it is not possible to transport the same volume of slag as natural aggregate. A similar situation occurs in the processing of minerals. The greater density and resistance of the slag, as well as the existence of heavy metals, makes the processing of these slightly more complex, requiring more energy and producing higher CO_2_ emissions.

According to the data shown in Figure 5, the processing of electric arc furnace slags to obtain road aggregates produces total emissions of 4.194 kg of CO_2_ equivalent, compared to 6.043 kg of natural siliceous aggregates. Therefore, there is a reduction in emissions due to the impact of global warming of approximately 30%. This is therefore a considerable environmental advantage.

In addition, the CML 2000 methodology permits the calculation of a series of other impacts. These impacts have been detailed, for the processing of natural siliceous aggregates and electric arc furnace slag, in Table 6.

Table 6 shows how in all impacts, emissions from the processing of natural siliceous aggregates are higher than from electric arc furnace slags, thus demonstrating the environmental advantages of using slags.

**Figure 6:**
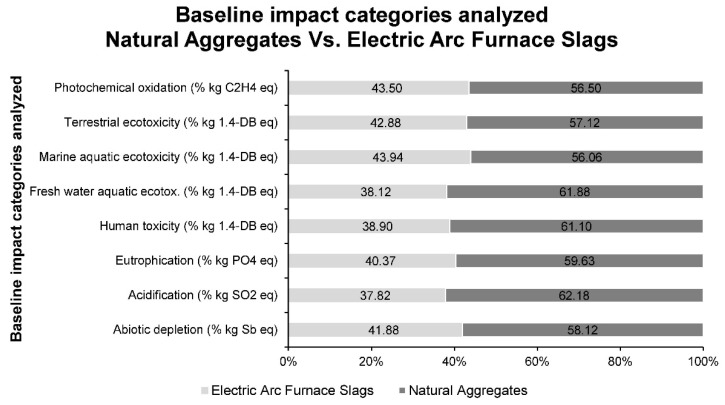
Percentages of emissions from various impacts for natural siliceous aggregates and electric arc furnace slags.

For a better understanding of the data in Table 5, Figure 6 shows the percentages that the processing of each material represents for each impact, taking as 100% the sum of both values.

Figure 6 shows how the greatest impact on emissions produced by the processing of electric arc furnace slag instead of the processing of natural siliceous aggregates is that of acidification. Next, and with similar values, are the impacts of fresh water aquatic ecotoxicity and human toxicity, which are very important factors to take into account for the well-being of the population and environmental safety. The impact that produces the least reduction is that of photochemical oxidation; however, all impacts produce lower emissions for slag than for natural aggregates.

In short, and according to the data reflected by the life cycle analysis, it can be stated that the use of electric arc furnace slags as coarse and fine aggregates in bituminous mixtures produces less environmental affliction on the environment.

## 4. Conclusions

The tests carried out in the methodology and life cycle analysis allow a series of partial conclusions to be drawn on the viability of using electric arc furnace slags as aggregates for bituminous mixtures. These conclusions are detailed below:

- The chemical composition of the slag reflects the composition of an inorganic material, the main chemical elements being calcium, iron, silicon, aluminum manganese and magnesium. The rest of the elements are available in low proportion. In addition, the main compounds are iron oxides, iron and manganese oxides and wollastonite, in smaller proportion. Therefore, the chemical composition shows a stable material without changes in shape or texture due to its contact with the environment.
- Electric arc furnace slags have a higher density than a conventional aggregate, greater than 3 t/m^3^. In turn, the shape of the particles makes the slag an ideal material for use in bituminous mixtures dedicated to high traffic roads, as has also been reflected in the sand equivalent test, (77 ± 2)%. The higher water absorption, WA_24_ = (3.33 ± 0.08)% conditions the absorption of a greater percentage of bitumen in the mixture, but does not imply a lower resistance to thermal fatigue.
- The high resistance to fragmentation of the electric arc furnace slags, (13 ± 1)%, and the resistance to thermal fatigue, (0.551 ± 0.016)% show the excellent quality of this material for use in bituminous mixtures. In addition, the polished stone value, 58 ± 1, shows the high resistance of the slag to polishing by the continuous passage of vehicles, maintaining the micro-texture of the aggregate throughout its working life.
- The concentrations of heavy metals in the leachate from electric arc furnace slags are lower than those set by Spanish regulations. Therefore, its use for bituminous mixtures is acceptable without producing environmental pollution, even more so when it is enveloped by bitumen, making it difficult to leach.
- The processing of electric arc furnace slags for use as aggregates for bituminous mixtures produces a reduction in environmental impact compared to the processing of natural siliceous aggregates of similar quality, with kg CO_2_ equivalent emissions of 4.194 and 6.043, respectively. It produces a reduction of 30% of kilograms of CO_2_ equivalent by using the slags; therefore, it is an environmentally friendly option.

According to the partial conclusions detailed above, based on the results of the methodology followed in the present investigation, it can be stated that the use of electric arc furnace slags as aggregates for bituminous mixtures, and even for high traffic roads, is feasible. In addition, its use reduces the deposition of this industrial by-product in landfills, reduces the extraction of raw materials, reduces the alteration of the landscape, and produces less environmental impact than the use of natural aggregates of similar quality.

## Figures and Tables

**Table 1:** Elemental analysis of the electric arc furnace slags.

Sample	Nitrogen, %	Carbon, %	Hydrogen, %	Sulfur, %
EAFS	0.005 ± 0.001	0.164 ± 0.003	0.044 ± 0.001	0.000 ± 0.001

**Table 2:** X-ray fluorescence of electric arc furnace slag.

Compound	wt, %	Est.Error
CaO	31.75	0.23
Fe_2_O_3_	21.96	0.21
SiO_2_	17.52	0.19
Al_2_O_3_	12.26	0.16
MnO	6.15	0.12
MgO	5.05	0.11
Cr_2_O_3_	2.73	0.08
TiO_2_	0.955	0.047
BaO	0.658	0.033
P_2_O_5_	0.319	0.016
SrO	0.186	0.0093
V_2_O_5_	0.159	0.0079
Nb_2_O_5_	0.0659	0.0033
S	0.0645	0.0032
ZrO_2_	0.0551	0.0028
K_2_O	0.0289	0.0016
CuO	0.0254	0.0017
ZnO	0.0245	0.0016
Co_3_O_4_	0.0147	0.0016
Eu_2_O_3_	0.0137	0.0065
WO_3_	0.0104	0.0031
Y_2_O_3_	0.0018	0.0005

**Table 3:** Physical properties of electric arc furnace slags.

Test	Standard	Value/Unit
Particle density (coarse aggregate), t/m^3^	UNE-EN 1097-6	3.13 ± 0.05
Particle density (fine aggregate), t/m^3^	UNE-EN 1097-6	3.34 ± 0.07
Water absorption WA_24_, %	UNE-EN 1097-6	3.33 ± 0.08
Sand Equivalent, %	UNE-EN 933-8	77 ± 2
Broken surfaces (coarse aggregate), %	UNE-EN 933-5	100 ± 1
Flakiness index, %	UNE-EN 933-3	0 ± 1

**Table 4:** Mechanical properties of electric arc furnace slags.

Test	Standard	Value/Unit
Resistance to fragmentation, %	UNE-EN 1097-2	13 ± 1
Resistance to freezing and thawing, %	UNE-EN 1367-1	0.551 ± 0.016
Polished stone value	UNE-EN 1097-8	58 ± 1

**Table 5:** Concentration of chemical elements in the leaching of the electric arc furnace slags.

Element	EAFS, mg/kg	Maximum Limits, mg/kg
Ba	2.132 ± 0.061	17.000
Cd	0.000 ± 0.001	0.009
Cr	0.237 ± 0.006	0.500
Mo	0.078 ± 0.002	0.500
Ni	0.004 ± 0.001	0.400
Pb	0.006 ± 0.001	0.500
Se	0.035 ± 0.001	0.100
V	1.175 ± 0.029	1.300
Zn	0.112 ± 0.003	1.200
As	0.000 ± 0.001	0.500
Cu	0.105 ± 0.003	2.000
Hg	0.000 ± 0.001	0.010
Sb	0.012 ± 0.001	0.060

**Table 6:** Impacts associated with the processing of natural siliceous aggregates and electric arc furnace slags for use in bituminous mixtures.

Impact Category	Unit	Natural Aggregate	EAFS
Abiotic depletion	kg Sb eq	0.043	0.031
Acidification	kg SO_2_ eq	0.033	0.020
Eutrophication	kg PO_4_ eq	0.011	0.007
Human toxicity	kg 1.4-DB eq	4.922	3.032
Fresh water aquatic ecotox	kg 1.4-DB eq	1.664	1.304
Marine aquatic ecotoxicity	kg 1.4-DB eq	3407.349	2557.654
Terrestrial ecotoxicity	kg 1.4-DB eq	0.017	0.013
Photochemical oxidation	kg C_2_H_4_ eq	0.002	0.001

## Data Availability

Data are contained within the article.

## References

[1] Yumashev A., Ślusarczyk B., Kondrashev S., Mikhaylov A. (2020). Global Indicators of Sustainable Development: Evaluation of the Influence of the Human Development Index on Consumption and Quality of Energy. Energies.

[2] Mohd Hasan M.R., You Z. (2015). Estimation of cumulative energy demand and green house gas emissions of ethanol foamed WMA using life cycle assessment analysis. Constr. Build. Mater..

[3] Guo C., Xu J., Yang L., Guo X., Liao J., Zheng X., Zhang Z., Chen X., Yang K., Wang M. (2019). Life cycle evaluation of greenhouse gas emissions of a highway tunnel: A case study in China. J. Clean. Prod..

[4] Menegaki M.E., Kaliampakos D.C. (2010). European aggregates production: Drivers, correlations and trends. Resour. Policy.

[5] Turk J., Mauko Pranjić A., Mladenovič A., Cotič Z., Jurjavčič P. (2016). Environmental comparison of two alternative road pavement rehabilitation techniques: Cold-in-place-recycling versus traditional reconstruction. J. Clean. Prod..

[6] Demirbas A. (2011). Waste management, waste resource facilities and waste conversion processes. Energy Convers. Manag..

[7] Alkins A.E., Lane B., Kazmierowski T. (2008). Sustainable Pavements. Transp. Res. Rec. J. Transp. Res. Board.

[8] Morseletto P. (2020). Targets for a circular economy. Resour. Conserv. Recycl..

[9] Anthonissen J., Van den bergh W., Braet J. (2016). Review and environmental impact assessment of green technologies for base courses in bituminous pavements. Environ. Impact Assess. Rev..

[10] Vaiana R., Balzano F., Iuele T., Gallelli V. (2019). Microtexture Performance of EAF Slags Used as Aggregate in Asphalt Mixes: A Comparative Study with Surface Properties of Natural Stones. Appl. Sci..

[11] Abousnina R., Manalo A., Ferdous W., Lokuge W., Benabed B., Saif Al-Jabri K. (2020). Characteristics, strength development and microstructure of cement mortar containing oil-contaminated sand. Constr. Build. Mater..

[12] Siddika A., Mamun M.A.A., Ferdous W., Saha A.K., Alyousef R. (2020). 3D-printed concrete: Applications, performance, and challenges. J. Sustain. Cem. Mater..

[13] Proctor D.M., Fehling K.A., Shay E.C., Wittenborn J.L., Green J.J., Avent C., Bigham R.D., Connolly M., Lee B., Shepker T.O. (2000). Physical and chemical characteristics of blast furnace, basic oxygen furnace, and electric arc furnace steel industry slags. Environ. Sci. Technol..

[14] Yildirim I.Z., Prezzi M. (2011). Chemical, mineralogical, and morphological properties of steel slag. Adv. Civ. Eng..

[15] Sideris K.K., Tassos C., Chatzopoulos A., Manita P. (2018). Mechanical characteristics and durability of self compacting concretes produced with ladle furnace slag. Constr. Build. Mater..

[16] Hosseini S., Soltani S.M., Fennell P.S., Choong T.S.Y., Aroua M.K. (2016). Production and applications of electric-arc-furnace slag as solid waste in environmental technologies: A review. Environ. Technol. Rev..

[17] Luz A.P., Tomba Martinez A.G., López F., Bonadia P., Pandolfelli V.C. (2018). Slag foaming practice in the steelmaking process. Ceram. Int..

[18] Sosa I., Thomas C., Polanco J.A., Setién J., Tamayo P. (2020). High Performance Self-Compacting Concrete with Electric Arc Furnace Slag Aggregate and Cupola Slag Powder. Appl. Sci..

[19] Lee J.-Y., Choi J.-S., Yuan T.-F., Yoon Y.-S., Mitchell D. (2019). Comparing Properties of Concrete Containing Electric Arc Furnace Slag and Granulated Blast Furnace Slag. Materials.

[20] Terrones-Saeta J.M., Suárez-Macías J., Iglesias-Godino F.J., Corpas-Iglesias F.A. (2021). Development of High Resistance Hot Mix Asphalt with Electric Arc Furnace Slag, Ladle Furnace Slag, and Cellulose Fibers from the Papermaking Industry. Appl. Sci..

[21] Terrones-Saeta J.M., Suárez-Macías J., Iglesias-Godino F.J., Corpas-Iglesias F.A. (2020). Evaluation of the Use of Electric Arc Furnace Slag and Ladle Furnace Slag in Stone Mastic Asphalt Mixes with Discarded Cellulose Fibers from the Papermaking Industry. Metals.

[22] Lizárraga J.M., Gallego J. (2020). Self-Healing Analysis of Half-Warm Asphalt Mixes Containing Electric Arc Furnace (EAF) Slag and Reclaimed Asphalt Pavement (RAP) Using a Novel Thermomechanical Healing Treatment. Materials.

[23] Terrones-Saeta J.M., Suárez-Macías J., Iglesias-Godino F.J., Corpas-Iglesias F.A. (2020). Development of Porous Asphalt with Bitumen Emulsion, Electric arc Furnace Slag and Cellulose Fibers for Medium Traffic Roads. Minerals.

[24] Skaf M., Pasquini E., Revilla-Cuesta V., Ortega-López V. (2019). Performance and Durability of Porous Asphalt Mixtures Manufactured Exclusively with Electric Steel Slags. Materials.

[25] Terrones-Saeta J.M., Suárez-Macías J., Iglesias-Godino F.J., Corpas-Iglesias F.A. (2020). Development of Slurry Surfacing with Electric Arc Furnace Slag for Pavements with Friction Problems. Minerals.

[26] Oluwasola E.A., Hainin M.R., Aziz M.M.A. (2015). Evaluation of asphalt mixtures incorporating electric arc furnace steel slag and copper mine tailings for road construction. Transp. Geotech..

[27] Zheng X., Easa S.M., Ji T., Jiang Z. (2020). Incorporating uncertainty into life-cycle sustainability assessment of pavement alternatives. J. Clean. Prod..

[28] Sas W., Głuchowski A., Radziemska M., Dzięcioł J., Szymański A. (2015). Environmental and Geotechnical Assessment of the Steel Slags as a Material for Road Structure. Materials.

[29] Jullien A., Proust C., Yazoghli-Marzouk O. (2019). LCA of alternative granular materials—Assessment of ecotoxicity and toxicty for road case studies. Constr. Build. Mater..

[30] Willms T., Echterhof T., Steinlechner S., Aula M., Abdelrahim A., Fabritius T., Mombelli D., Mapelli C., Preiss S. (2020). Investigation on the Chemical and Thermal Behavior of Recycling Agglomerates from EAF by-Products. Appl. Sci..

